# Microbial and Biochemical Analyses of High-Quality, Long-Ripened, Blue-Veined Cabrales Cheese

**DOI:** 10.3390/foods14132366

**Published:** 2025-07-03

**Authors:** Javier Rodríguez, Paula Rosa Suárez, Souvik Das, Lucía Vázquez, Sonam Lama, Ana Belén Flórez, Jyoti Prakash Tamang, Baltasar Mayo

**Affiliations:** 1 Departamento de Microbiología y Bioquímica, Instituto de Productos Lácteos de Asturias (IPLA), Consejo Superior de Investigaciones Científicas (CSIC), Francisco Pintado Fe, 26, 33011 Oviedo, Spain; javier.rodriguez@ipla.csic.es (J.R.); paula.suarez@ipla.csic.es (P.R.S.); lucia.vazquez@ipla.csic.es (L.V.); abflorez@ipla.csic.es (A.B.F.); 2 Instituto de Investigación Sanitaria del Principado de Asturias (ISPA), Avenida de Roma s/n, 33011 Oviedo, Spain; 3 Department of Microbiology, School of Life Sciences, Sikkim University, Science Building, Dara Goan, Tadong, Gangtok 737102, India; svkstpp95@gmail.com (S.D.); sonam.lama6233@gmail.com (S.L.); jyoti_tamang@hotmail.com (J.P.T.)

**Keywords:** traditional cheeses, Cabrales cheese, cheese microbiota, cheese biochemistry, starters, lactic acid bacteria

## Abstract

Sixteen long-ripened, high-quality Cabrales cheeses from independent producers underwent a comprehensive biochemical and microbiological characterisation. Significant variations in total microbial counts and specific microbial groups were observed among the cheeses. A metataxonomic analysis identified 249 prokaryotic amplicon sequence variants (ASVs) and 99 eukaryotic ASVs, respectively, which were classified into 52 prokaryotic and 43 eukaryotic species. The predominant species included bacteria of the genera *Tetragenococcus*, *Lactococcus* (of which *Lactococcus lactis* was used as a starter), and *Staphylococcus*, followed by *Brevibacterium* and *Corynebacterium* species. The starter mould *Penicillium roqueforti* was highly abundant in all cheeses; *Debaryomyces hansenii*, *Geotrichum candidum*, and *Kluyveromyces* spp. constituted the subdominant fungal populations. Glutamic acid (≈20 mg g^−1^) was the most abundant free amino acid in all samples, followed by lysine, leucine, and valine (≈10–13 mg g^−1^). Moderate-to-high amounts of the biogenic amines tyramine and ornithine were detected. A large variation between cheeses of the main organic acids (lactic, acetic, or butyric) was detected. Differences between samples were also observed for the majority volatile compounds, which included organic acids, alcohols, esters, and ketones. Positive and negative correlations between bacterial and fungal species were detected, as well as between microbial populations and key biochemical markers. Among the latter, *Tetragenococcus halophilus* correlated positively with ethyl caprylate and hexanoic acid, and *Loigolactobacillus rennini* correlated positively with γ-aminobutyric acid. Conversely, *Staphylococcus equorum* showed a strong negative correlation with ethyl caprylate and capric acid. These microbial and biochemical insights enabled us to propose a microbiota-based starter culture comprising prokaryotic and eukaryotic components to enhance Cabrales cheese quality.

## 1. Introduction

Recent advances in next-generation sequencing (NGS), new metabolomic analysis, and state-of-the-art sophisticated bioinformatic tools have given rise to the ‘cheesomics’ science [1], which has been shown to provide much deeper insights into cheese microbial diversity [2,3] and succession [4] than ever gleaned from culturing. These methods may further supply information on the potential functionality of cheese-associated microorganisms [5,6]. In addition, biochemical analyses can detect and quantify a myriad of chemical biomarkers of microbial origin in cheese, such as free amino acids, fatty acids, and volatile compounds (VOCs) [7,8], any of which might contribute to a cheese’s texture, taste, and aroma properties. The finding of correlations between microbial populations and biochemical variables might provide pivotal information on the origin, diversity, and function of specific components of the cheese microbiota [1,8,9]. The microbiota of cheeses is also of interest for economic and safety reasons; spoilage microorganisms can downgrade cheese quality, leading to economic losses, and the growth of zoonotic pathogens (e.g., *Listeria monocytogenes* and *Staphylococcus aureus*) can cause infections or intoxications [10]. Better knowledge of the cheese microbiota could, therefore, help control the manufacturing and ripening processes and, thus, increase the overall cheese quality [11].

The use of different NGS techniques has revealed traditional cheeses, particularly those made from raw milk, to possess an unprecedented diversity of microbes [2,3,4,5,7,8,12,13]. However, the significance of their presence to cheese technology, quality, and safety is largely unknown [2,13]. After their detection, for proper characterisation, cheese-borne organisms should be recovered in culture by conventional or novel cultivation (‘culturomics’) methods [14]. Characterised strains that are harmless and show desirable traits might then be exploited as starter and/or adjunct cultures to ensure the typical bouquet of those cheeses to which they are native, or to diversify flavour in other varieties.

Cabrales is a traditional, blue-veined cheese manufactured in Asturias (northern Spain) that has enjoyed protected designation of origin (PDO) since 1981. It is made from raw cow’s milk supplemented, if available, with variable mixtures of sheep’s and goat’s milk (https://www.quesocabrales.org/pdf/pliego-condiciones-dop-cabrales.pdf, accessed on 1 May 2025). The production of Cabrales cheese involves curdling a mixture of evening and morning milk at 28–30 °C using animal rennet. The curd is then cut into hazelnut-sized pieces and placed in cylindrical molds to drain the whey, without applying pressure. After 48 h at room temperature—during which the cheeses are turned several times—they are covered with coarse salt and left at room temperature for an additional 10–15 days. Finally, the cheeses are transferred to natural caves within the production area, where they ripen at a nearly constant temperature (9–12 °C) and high relative humidity (90–95%) [15,16]. Until recently, Cabrales was manufactured without the addition of any starter or ripening culture, although nowadays the addition of a native starter composed of *Lactococcus lactis* strains and commercial *Penicillium roqueforti* spores is common practice. Whether manufactured with or without such starters, a large number of different microbial populations develop and succeed one another during manufacturing and ripening, both in the surface (rind) and interior (core) of the cheese [15,16]. NGS techniques have recently identified in Cabrales many bacterial species that have gone undetected by culturing [17]. Such discovery has led to the isolation of *Tetragenococcus* species from mature cheeses [18].

The present work aimed to further examine the microbial diversity of Cabrales cheese by an NGS technique in terms of its bacterial and fungal populations, detect metabolites and VOCs of microbial origin, and seek correlations between the microorganisms themselves and between microorganisms and biochemical markers. The goal was to identify microorganisms contributing to the sensory properties of the cheese that may serve as a complex, microbiota-based starter to enhance cheese quality.

## 2. Materials and Methods

### 2.1. Cheese Sampling and Microbial Analyses

In this work, 16 mature Cabrales cheeses, made by independent manufacturers and presented at the 50th Cabrales Cheese Contest (August, 2022), were sampled and analysed. The type of milk used for manufacture (cow or mixtures of cow, ewe, and goat) and the ripening time of the cheeses (which might range from 6 to 12 months) was not available. Cheeses were evaluated by a trained panel of ten people and ordered for sampling in a descending order by the score they received from a total of 1260 possible points, C1 (1129 points) to C16 (654 points). For some statistical analyses the cheeses were arbitrarily grouped into three sensory classes, A (C1 through C6, from 1129 to 1000 points), B (C7 though C13, from 962 to 858 points), and C (C14 through C16, from 792 to 654 points).

Wedges of cheese weighing approximately 250 g were taken and transported to the laboratory under refrigerated conditions. Following FIL-IDF standard 50B [19], 10 g samples from the cheese core were homogenised with 90 mL of a 2% (*w*/*v*) sodium citrate solution at 45 °C in a Colworth Stomacher 400 (Seward Ltd., London, UK). Ten-fold serial dilutions were carried out in Ringer’s solution (Merck, Darmstadt, Germany) and plated in duplicate (two plates per dilution) onto non-selective and selective media, as follows: total aerobic mesophilic bacteria on Plate Count Milk Agar (PCMA) (Merck), enumerating after 72 h of incubation at 32 °C; lactococci on M17 agar (Formedium, Norfolk, UK) supplemented with glucose (0.5%) (GM17A), enumerating after 48 h of incubation at 32 °C; lactobacilli on de Man, Rogosa, and Sharpe agar (MRSA) (Merck), adjusted to pH 5.4, enumerating after 48 h of incubation at 32 °C; enterococci on Slanetz and Bartley agar (S-BA) (Merck), enumerating after 24 h of incubation at 42 °C; enterobacteria and coliforms on Violet Red Bile Glucose agar (VRBGA) (Merck) and Violet Red Bile Lactose agar (VRBLA) (Merck), respectively, using the pour-plate and overlay technique and enumerating after 24 h of incubation at 37 °C; micrococci and staphylococci on Baird-Parker agar (B-PA) (Merck) supplemented with egg yolk tellurite solution (Biokar Diagnostics, Allonne, France), enumerating black colonies with or without egg yolk clearing after 24 h of incubation at 37 °C; and yeasts and moulds on Yeast-Extract Glucose Chloramphenicol agar (YGCA) (Merck), enumerating after 3–5 days of incubation at 25 °C.

### 2.2. Metataxonomic Analysis

#### 2.2.1. Isolation of Total Microbial DNA from Cheese Microbes

Cheese samples (5 g from the core) were homogenised with 45 mL of 2% (*w*/*v*) sodium citrate solution at 45 °C in a Colworth Stomacher 400. After centrifugation at 10,000 rpm for 10 min at 4 °C, the top fat layer was removed using a sterile cotton swab, the supernatant was discarded, and the microbial pellet was used for total DNA extraction using a Food-Extract DNA Purification Kit (EURx, Gdańsk, Poland), according to the manufacturer’s instructions with the following modifications: the commercial lysis buffer Res FE was supplemented with 20 mg mL^−1^ lysozyme (Merck), 25 U mutanolysin (Sigma-Aldrich, Saint Louis, MO, USA), and 10 µg of lysostaphin (Sigma-Aldrich). Cell suspensions were incubated at 37 °C for 45 min, and then at 55 °C for 15 min. Incubated cells were then subjected to mechanical lysis with 0.5–1.0 mm crystal beads (BeadTubeDry; EURx) using a FastPrepFP120 Cell Disrupter (Qbiogene, Carlsbad, CA, USA) at 5.5 m s^−1^ for 30 s. The DNA was purified using a Food-Extract DNA Purification Kit according to the recommendations. Finally, DNA was quantified fluorometrically using a Qubit 4.0 fluorometer (Invitrogen, Carlsbad, CA, USA) and a Qubit 1 X dsDNA BR Assay Kit (Invitrogen, Walthman, MA, USA). DNA quality was assessed by measuring the A260/230 nm and A260/280 nm absorbance ratios using a Genova Bio UV–visible spectrophotometer (Jenway, Staffordshire, UK). Purified DNA was stored until processing at −20 °C.

#### 2.2.2. Amplification and Sequencing of Ribosomal Sequences

Segments of ~445 bp of the prokaryotic 16S rRNA gene (V3-V4 hypervariable region), and ~315 bp of the fungal internal transcribed spacer 2 (ITS2) of the ribosomal region were independently amplified by a polymerase chain reaction (PCR) and sequenced. These regions were amplified, with primer pairs 16S_f (5′-TACGGGAGGCAGCAG-3′) and 16S_r (5′-CCAGGGTATCTAATCC-3′) [20], and ITS2_f (5′-GCATCGATGAAGAACGCAGC-3′) and ITS2_r (5′-TCCTCCGCTTATTGATATGC-3′) [21], respectively, and using previously reported amplification conditions. Amplicons were pair-end-sequenced using an Illumina platform at Eurofin Genomics (Ebersberg, Germany). More than 60,000 raw amplicon sequences (read pairs) were obtained per sample. Illumina adapters were removed with the CutAdapt program [22] and the reads filtered for quality (Q30) using FASTQ [23].

Quality-checked and cleaned read sequences were analysed using QIIME2 (v.2023.2, http://qiime.org/ accessed on 20 October 2024) [24]. Sequences were demultiplexed, denoised, and merged using the QIIME2 q2-dada2 plugin [25]. The INVIEW Microbiome Profiling 3.0 (Eurofins Genomics) was used for initial taxonomic analysis. The best matching taxonomic reference sequence with >99% identity was then added to unique representative sequences after comparing to those in the DAIRYdb (https://github.com/marcomeola/DAIRYdb accessed on 25 October 2024) [26] and UNITE fungal internal transcribed spacer (ITS) (https://unite.ut.ee/ accessed on 30 October 2024) [27] databases using the QIIME2 classifier. Contaminant mitochondrial and chloroplast sequences were removed using the QIIME2 filter-table and filter-seq scripts of the taxa plug-in. Similarly, amplicon sequence variants (ASVs) with <10 reads for the total of all samples were removed. To verify genus and species assignments, representative sequences of each ASV were compared against those in the NCBI database using the BLAST+ tool version 2.16.0 (http://www.ncbi.nlm.nih.gov/blast/; accessed on 14 December 2024).

### 2.3. Metabolic Analysis of Cheeses

#### 2.3.1. Organic Acids and Sugars

Organic acids and sugars were extracted from the cheese samples and determined by HPLC following the method of Alegría et al. [28]. Briefly, organic acids and sugars were separated using an ICSep ICE-ION-300 ion-exchange column (ThermoFisher, Waltham, MA, USA), employing an 8.5 mM H_2_SO_4_ aqueous mobile phase, at an operating temperature of 65 °C and a flow rate of 0.4 mL min^−1^. Organic acids were identified using a 996 Photodiode Array Detector (Waters, Milford, MA, USA) at 210 nm, and sugars were identified using a 410 Differential Refractometer (Waters) at 280 nm. Quantification was performed using calibration curves prepared with commercial standards of lactose, glucose, galactose, and a range of organic acids (all from Sigma-Aldrich).

#### 2.3.2. Amino Acids and Biogenic Amines

The amino acid and amino-acid-derived compounds in cheese were extracted, derivatised with diethyl ethoxymethylenemalonate (DEEMM), and analysed by ultra-high-performance liquid chromatography (UHPLC), following the procedure by Redruello et al. [29]. Cheese processing, deproteinisation, defatting, amino acid extraction, derivatisation, detection, and quantification were performed as previously described [29].

#### 2.3.3. Volatile Compounds (VOCs)

VOCs in cheese were determined following a solid-phase microextraction gas-chromatography (SPME-GC) method as reported by Walsh et al. [9]. Briefly, samples of 4 g of finely grated cheese were placed into 20 mL screw-capped SPME vials (Agilent Technologies, Santa Clara, CA, USA), which were then sealed with a PTFE/silicone liner septum and equilibrated at 40 °C for 10 min with pulsed agitation for 5 s at 500 rpm using a PAL RSI 120 device (CTC Analytics, Zwingen, Switzerland). VOCs were absorbed onto an ARR11-DVB-120/20 DVB/PDMS fibre (CTC Technologies) exposed to the headspace above the samples for 20 min at a depth of 40 mm and at 60 °C. Eluted compounds were identified based on their retention times and by comparison of their mass spectra in the Wiley Mass Spectral database (Wiley and Sons, NY, USA); the match score was set at >700. Quantification was performed using a GC flame ionisation detector (FID) HP5890 series II plus (Agilent).

### 2.4. Statistical Analysis

Diversity indices, Good’s coverage, Shannon’s H, and Simpson’s D values were calculated using the PAST (Paleontological Statistics Software Package) software v.4.0 [30]. All other statistical analyses were performed and network graphics were produced using the PAST software and Cytoscape software v.3.10 [31]. Biplots were created using the PAST software v.4.0. Correlation analyses were conducted for sequence and analyte data. Spearman’s correlations were calculated using the PAST software v.4.0; significance was set at *p* < 0.05. The interquartile range (IQR) method was applied to detect outliers in the datasets of organic acids, sugars, amino acids, amines, and volatile compounds. As reported elsewhere [32], values below Q1–1.5×IQR or above Q3 + 1.5×IQR were considered outliers.

A principal component analysis (PCA) was performed using the complete metabolite data set (free amino acids, organic acids, and volatile compounds). To apply this statistical exploratory technique, all numeric features were re-scaled using standard normalisation and transforming the data values to a mean of 0 and a standard deviation of 1. The covariance matrix was then computed to capture feature relationships and its eigenvectors (principal components) and eigenvalues (explained variance) were determined. Correlations between features and principal components were calculated and the biplots created using Python v. 3.12.7 and the Scikit-learn library [33].

## 3. Results

### 3.1. Microbial Counts

Large differences in total microbial counts and several microbial groups were recorded among the 16 cheeses (Table 1). Depending on the cheese, either bacterial or fungal populations reached the highest numbers. Presumptive lactococci and streptococci (which ranged from 2.30 to 7.16 log_10_ CFU g^−1^) or yeasts and moulds (which ranged from 2.60 to 7.31 log), were the majority populations in most cheeses. At 4.0–6.0 log, presumptive *Lactobacillaceae*, enterococci, and micrococci and staphylococci alternated as the subdominant populations. *Enterobacteriaceae* and coliforms were below the limit of detection (2 log_10_ CFU g^−1^) in all samples. Intriguingly, in repeated analyses, cheese C5 returned counts for all microbial groups at either the limit of detection or below.

### 3.2. Molecular Microbiology

The rarefaction curves for the sequenced amplicons (16S rDNA and ITS2) proved to be close to or on the saturation plateau, suggesting adequate sequencing depth. Alpha-diversity measures, such as Good’s coverage (0.98 ± 0.12 and 0.97 ± 0.05), Shannon’s H (5.38 ± 0.23 and 5.67 ± 0.02), and Simpson’s D (0.90 ± 0.02 and 0.89 ± 0.20) values for 16S rDNA amplicons (bacteria) and ITS amplicon sequences (fungi), indicated good coverage of the microbial diversity for each sample. Large differences in the relative abundance of reads for several prokaryotic and eukaryotic populations between the cheeses were noted.

**Figure 1:**
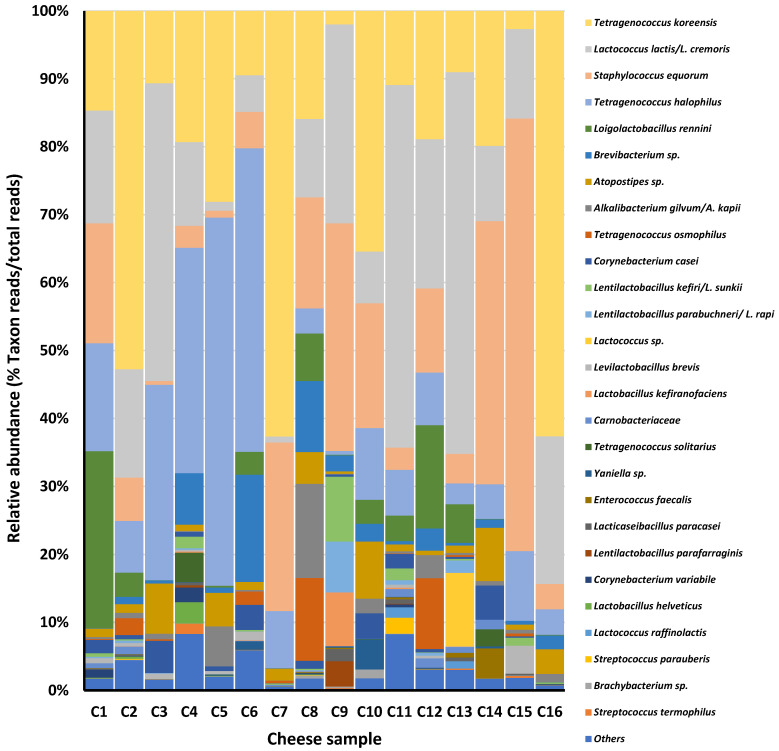
Distribution and relative abundance of bacterial taxons in the 16 Cabrales cheese samples of this study, as identified, mostly at the species level, by 16S rDNA amplification, sequencing, and sequence analysis. Only taxons showing a relative abundance >1% are depicted.

Analysis of the 16S rDNA amplicons revealed 249 amplicon sequence variants (ASVs) with a mean length of 422.98 bp of merged sequences. Sequences were assigned to 52 bacterial phylotypes (species- or genus-like taxa), each represented by a range of 1–11 ASV. A total of 29 out of the 52 phylotypes (10–24 phylotypes per sample) showed a relative abundance of >1%, representing a coverage of 94.4–99.7% of the diversity per sample (Figure 1). Overall, 16 prokaryotic phylotypes were present in at least 50% of the samples, ranging in relative abundance from 80.0% to 99.5% of the reads. Only six were shared by all cheeses (relative abundance 70.9–99.0%).

Although significantly different in relative abundance among the cheeses, the most prevalent bacterial species were *Tetragenococcus koreensis* (2.0–62.7%; mean 23.4%), *Lactococcus lactis/L. cremoris* (0.9–56.2%; mean 20.1%), *Staphylococcus equorum* (0.6–63.6%; mean 15.8%), and *Tetragenococcus halophilus* (3.1–54.2%; mean 15.3%). Subdominant species included *Brevibacterium* spp. (0.1–15.8%; mean 3.1%), *Atopostipes* spp. (0.4–8.4%; mean 2.9%), *Alkalibacterium gilvum/A. kapii* (0.0–13.8%; mean 1.9%), *Tetragenococcus osmophilus* (0.0–12.2%; mean 1.8%), *Corynebacterium* spp. (0.0–5%; mean 1.6%), and *Lentilactobacillus* spp. (0.0–17.0%; mean 1.7%). Several species from the former *Lactobacillus* genus appeared at low levels in most samples. Noteworthy, *Loigolactobacillus rennini* reads (0.0–26.0%; mean 4.3%) were found at high relative numbers in a few cheeses. Certain species were associated, at high relative abundance, with a single cheese, such as *Lactococcus raffinolactis/L. garviae/L. laudensis* (10.8% in C13), *Lactobacillus kefiranofaciens* (7.9% in C9), *Yaniella* spp. (4.4% in C10), *Enterococcus faecalis* (4.4% in C14), *Tetragenococcus solitarius* (4.3% in C4), *Levilactobacillus brevis* (4.1% in C15), and *Lentilactobacillus parafarraginis* (3.7% in C9).

**Figure 2:**
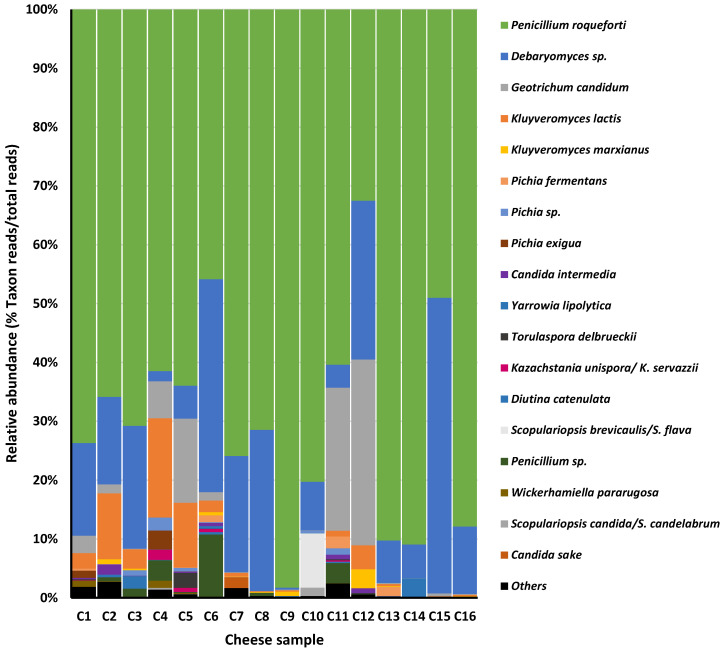
Distribution and relative abundance of fungal taxons in the 16 Cabrales cheese samples of this study, as identified by ITS amplification, sequencing, and sequence analysis. Only taxons showing a relative abundance >1% are depicted.

For the eukaryotic communities, 99 ASVs were identified with an average sequence length of 313.02 pb of merged sequences. Sequences were assigned to 43 different fungal phylotypes, of which only 18 (with a range of 4–12 taxa per sample) showed a relative abundance of >1%. The phylotypes were composed of 1–21 different ASVs (with the *Penicillium roqueforti/P. carneum* phylotype having 21). Six eukaryotic phylotypes were observed in at least half of the cheeses with a relative abundance of 88.7–99.9%; only two of these were present in all cheeses (relative abundance of 59.5–99.4%). The two taxa detected in all cheeses were the mould *P. roqueforti* (used as a starter) and the yeast *Debaryomyces hansenii* (Figure 2). Despite this, the prevalence of *P. roqueforti* in the different cheeses was diverse, from 32.5–90.9%. Wide relative abundance was also recorded for *D. hansenii* (0.3–50.2%), whose numbers were surpassed in certain cheeses by other yeast species, such as *Geotrichum candidum* (*Galactomyces candidus*) (in C11 and C12; 24.3% and 31.5%, respectively) and *Kluyveromyces lactis* (in C4 and C5; 16.8.0% and 11.0%, respectively). In addition, the *Scopulariopsis brevicaulis/S. flava* phylotype showed a fairly high relative abundance of 9.2% in cheese C10. Several yeast species, such as *Kluyveromyces marxianus, Pichia fermentans, Pichia exigua, Yarrowia lipolytica, Torulaspora delbrueckii*, and *Diutina catenulata*, appeared in certain cheeses in lower percentages (1–3%).

### 3.3. Analysis of Cheese Metabolites

A certain diversity of organic acids, sugars, and other compounds was detected across the different cheeses (Table 2). High concentrations of ammonia (1505–5104 mg 100 g^−1^) were scored for all cheeses; in contrast, low (and similar) concentrations of orotic, pyruvic, and uric acids were recorded for all samples. Wide variability across the different cheeses was detected for acetic (23–390 mg 100 g^−1^), butyric (66–454 mg 100 g^−1^), and lactic (14–422 mg 100 g^−1^) acids, as well as for lactose (421–714 mg 100 g^−1^). Formic acid, galactose, and glucose were detected in only two, four, and fourteen cheese samples, respectively, and only in small quantities.

**Figure 3:**
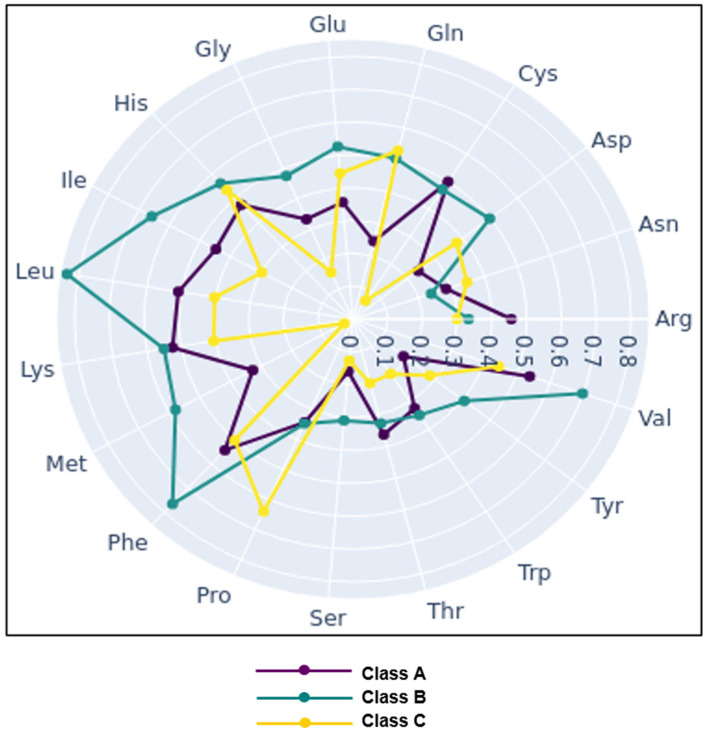
Graphical representation of the normalised averages of the free amino acid content of the three classes (A, B, and C) defined from the highest to the lowest scores obtained by the cheeses during their sensory evaluation.

Large differences in free amino acids were seen between the samples (Table S1). The interquartile range (IQR) analysis showed a large statistical dispersion of the concentration values of some amino acids. Glutamic acid was the amino acid in the highest concentration in most cheeses (>1500 mg 100 g^−1^), followed by lysine and leucine (≈1000–1300 mg 100 g^−1^). Cysteine followed by asparagine and proline were the least abundant free amino acids in all cheeses (0–123 mg 100 g^−1^). The graphical representation of the normalised averages of the free amino acid content within each of the three arbitrary sensory classes showed that cheese quality was related to a balanced content of the different free amino acids rather than to the total amount (Figure 3). Amino-acid-derived compounds, including biogenic amines and compounds resulting from the decarboxylation of amino acids, were also very variable (Table 3). Ethylamine and agmatine were found at low levels in two and six cheeses, respectively. Histamine and tyramine, the most toxic amines, ranged widely from 0–85 and 130–513 mg 100 g^−1^, respectively. High concentrations of ornithine and tryptamine were detected in all cheese samples. For other related compounds, lower concentrations were scored, except for γ-aminobutyric acid (GABA) in two samples.

SPME-GC identified 109 VOCs, including acids, aldehydes, alcohols, esters, ketones, aromatic, and other compounds (Table S2). Of these, 16 were found in all cheeses, 9 in the majority, 14 in only two samples, and 37 in only one. Butanoic, hexanoic, caprylic, capric, and dodecanoic acids, 2-heptanol and 2-nonanol alcohols, hexanoic octanoic acid-ethyl and ethyl caprylate esters, and 2-heptanone, 2-nonanone, and 8-nonen-2-one ketones were the majority VOCs. Except for butanoic acid, which was absent from three samples, these compounds were found in all cheeses, although their concentrations differed.

**Figure 4:**
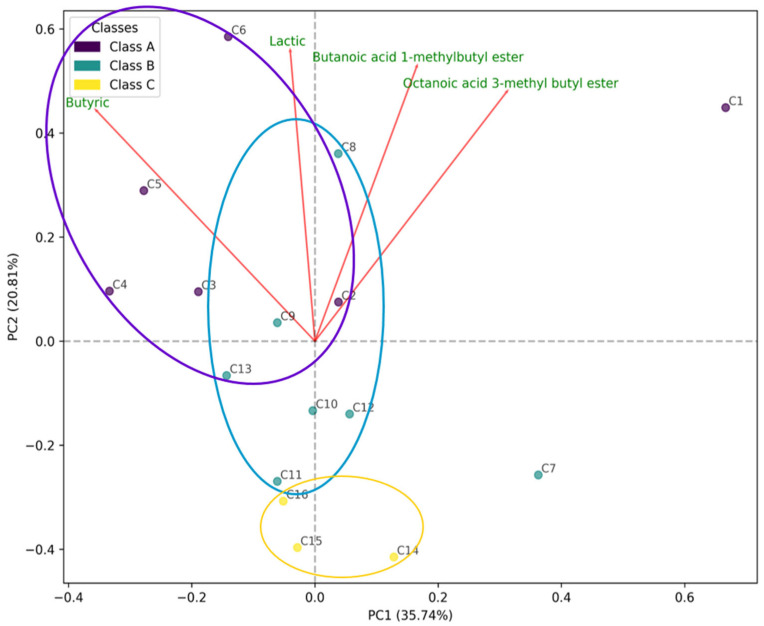
Principal component analysis (PCA) and score plot of the Cabrales metabolites, including organic acids, amino acids, and volatile compounds, in the three classes (A, B, and C) defined from the highest to the lowest scores obtained by cheeses during their sensory evaluation. Butyric acid, lactic acid, butanoic acid-1-methyl butyl ester, and octanoic acid 3-methyl butyl ester were the compounds contributing the most to separate the cheeses by the PCA test. Feature vector analysis (red arrows) suggested butyric and lactic acids as compounds leading to cheese quality.

A principal component analysis (PCA) was carried out using all metabolites identified in the cheeses of this study (137 chemical compounds) (Figure 4). Only 56% of the variance in the data set was explained by the two principal components, PC1 and PC2. Nonetheless, these two components grouped a majority of the cheeses into the three established sensory classes (A, B, and C). Class A was found to be the most diverse, while class C was the most compact and with a greater distinctness. The compounds butyric acid and lactic acid, followed by butanoic acid-1-methyl butyl ester and octanoic acid 3-methyl butyl were the main drivers contributing to separate the cheeses by the PCA. Octanoic acid influenced the separation over PC1, while butanoic ester was found to be prominent on both PC1 and PC2. Lactic acid contributed to separate along PC2, and butyric acid proved to be positive along PC2 but negative on PC1. Lactic acid and butyric acid influenced class A the most (sample C1, sample C5, sample C6), whereas class B (samples C7, C8, and C9) was impacted by butanoic and octanoic esters in a moderate manner. Class C samples showed the least association with these compounds. Feature vector analysis of butyric and lactic acids, whose increased concentrations point towards class A cheeses, suggests these compounds may lead to Cabrales cheese quality.

### 3.4. Correlations Between Microorganisms and Metabolites in Cabrales Cheese

**Figure 5:**
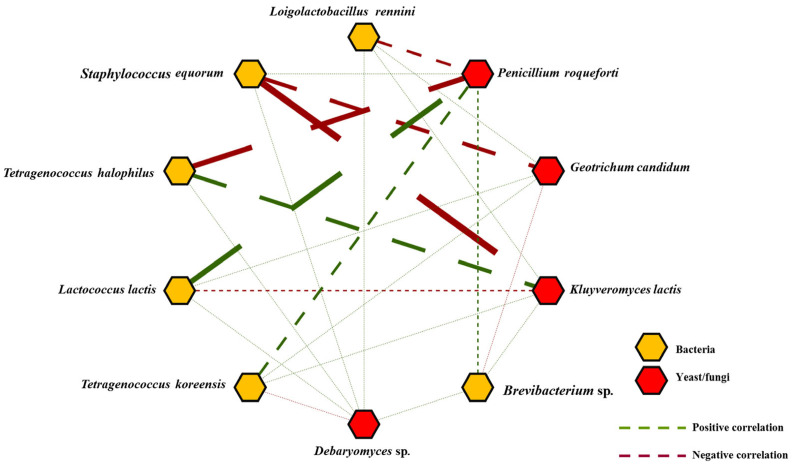
Positive and negative Person’s correlations in Cabrales cheese between bacterial and fungal populations (*p* < 0.05). Only microbial members with a relative abundance >1% were taken into consideration.

Positive and negative interspecies correlations with themselves and with each other were detected between several prokaryotic and eukaryotic communities. As an example, Figure 5 shows the significant correlations between the bacterial and fungal populations. Strong direct correlation was seen between *L. lactis* and *P. roqueforti*, indicating the co-occurrence of these microbes, although smaller, direct correlations were also noted between *T. halophilus* and *T. koreensis* and between *K. lactis* and *P. roqueforti*. Strong inverse correlations were shown between *S. equorum* and *K. lactis* and *G. candidum*, and *T. halophilus* and *P. roqueforti*. *P. roqueforti* also showed an inverse correlation with *L. rennini*.

**Figure 6:**
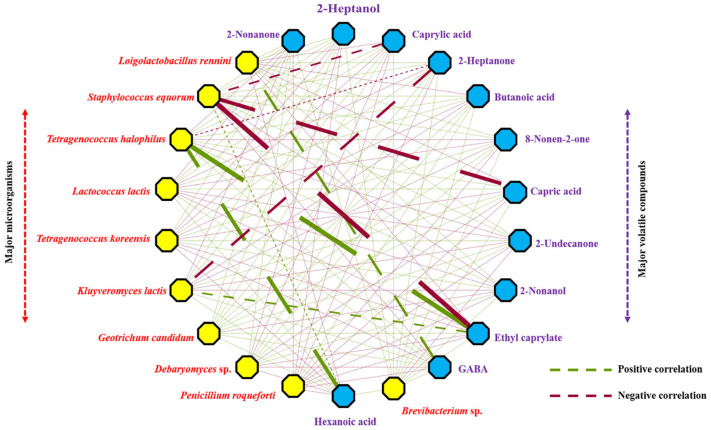
Network analysis signifying positive and negative Person’s correlations between majority microbial populations and majority metabolites in Cabrales cheese (*p* < 0.05).

A correlation analysis between cheese metabolites and the majority of the microbial populations was undertaken to assess their possible cause–effect relationships (Figure 6). This analysis showed *T. halophilus* to have a strong positive correlation with the presence of ethyl caprylate and hexanoic acid VOCs, while *L. rennini* correlated in the same way with GABA. In contrast, a strong inverse correlation was detected between *S. equorum* and ethyl caprylate and capric and caprylic acids. *K. lactis* was inversely associated with 2-heptanone. A myriad of other minor direct and inverse correlations were also detected between species and the examined analytes.

## 4. Discussion

The total microbial and group-specific counts of the 16 cheeses of this study were similar to those reported for other blue-veined cheeses, such as Blue D’Auverne [34] and Valdeón [35], but they were lower than those noted for Gorgonzola and other blue varieties [36]. Yeasts and moulds made up the majority of microbial populations in most of the studied cheeses, which agrees well with the continuous reduction in LAB and the development of fungal populations reported during blue cheese ripening [36]. Notably, *Enterobacteriaceae* and coliforms were absent from all the present samples. These groups are widely recognised as indicative of microbial contamination and are associated with the potential presence of frank pathogens.

A large number of reads of the halotolerant, alkalophilic *Tetragenococcus* and *Staphylococcus* species has recently been reported for Cabrales cheese [17] and in some other long-ripened cheeses [4,37]. *Tetragenococcus* spp. and *S. equorum* have been recently identified in seven commercial rennet samples, and the latter has occasionally been recovered from salt [37]. Through transfer via curds, these bacteria could colonize brines [37,38] and ripening shelves [17], which thus become secondary inoculation sources. The presence of genes and operons involved in lactose utilisation in the genome of cheese strains of *Tetragenococcus* strongly suggests the long-standing adaptation of these bacteria to the dairy environment [18]. Recent characterisations of *T. koreensis* and *T. halophilus* isolated from Cabrales and Picón Bejes-Tresviso cheeses proved them to be safe and to have enough genetic potential to encode enzymatic profiles similar to those of LAB [18]. The same goes for *S. equorum*, a species currently used as a starter for surface-ripened cheeses [39]. In one of the present cheeses, isolates of the *Staphylococcus carnosus* group were dominant and showed co-exclusion with *S. equorum*. Strains of these two groups might have similar biochemical profiles and thus compete with each other for nutrients. The development of *Staphylococcus* species in cheese has proven to be stimulated by *Penicillium* and *Scopulariopsis* fungi [40]. Despite all this, the relationship of both *Tetragenococcus* spp. and *S. equorum* populations with the Cabrales cheese quality has yet to be determined. For instance, *Tetragenococcus* proved to correlate with ethyl caprylate, but whether the compound is pivotal in the Cabrales aroma or it is produced or facilitated by species of this genus is currently not known. However, a major relative abundance of *Tetragenococcus* reads with cheeses of Class A was observed (Figure 1), which suggests they might contribute to the Cabrales sensory profiles. The role of *Tetragenococcus* and *Staphylococcus* species should be tested in experimental trials by using properly characterised strains as adjunct cultures.

*Corynebacterium* spp. and *Brevibacterium* spp. reads were commonly retrieved from the present cheeses. These two genera belong to the phylum *Actinomycetota*, produce aroma and carotenoid compounds, and are pivotal in the ripening of smear-ripened cheeses [13]. The surface of Cabrales resembles that of smear-ripened cheeses. However, the large size of the cheese (2.7–3.0 kg) may limit the effect of the rind microbiota in the cheese matrix. Uncommon bacterial and fungal microbes have been reported in many metagenomic studies in cheese [4,5,7,41]. The recovery of these microbes via culturing is currently being addressed, which would lead to their biochemical, genetic, and genomic characterisation. This might provide clues on their potential influence on the quality and/or safety of the cheese.

As in many other metataxonomic studies [5,42,43], only a small number of reads for genera and species of opportunistic (e.g., *Serratia* spp. and pyogenic streptococci) and true pathogens (e.g., *Escherichia coli*, *Shigella* spp.) were detected by the microbial fingerprinting of Cabrales cheese. Of these, coliforms have been reported to be abundant during manufacturing [16]. As for yeasts and moulds, only a few reads of *Candida albicans* and *Candida parapsilosis* sequences were scored in a single cheese each, and *Pichia kudriavzevii* reads were scored in seven cheeses. These yeasts rank, respectively, among the critical-, high-, and medium-priority groups of fungal pathogens [44]. Despite being made from raw milk, the current molecular results point towards long-ripened Cabrales cheese being safe from a microbiological viewpoint. However, except for *S. aureus*, the presence in the cheese samples of viable pathogens of bacterial and fungal origin was not evaluated.

Notable numerical differences were seen between the relative abundance of reads in the metataxonomic analysis and the culturing numbers of some microbial groups (Table 1). It is worth mentioning the low numbers of LAB populations in some cheese samples, populations that had already been reported in great abundance at several ripening stages (up to 60 days) during the Cabrales manufacturing process [15,16]. This suggests that the long ripening caused microbial groups to die or enter into a viable but non-cultivable (VBNC) state. Death and lysis of the cells release proteases, peptidases, lipases, and other degrading enzymes of ripening into the cheese matrix, which speeds up the production of aroma and taste compounds [45]. Conversely, VBNC or dead microorganisms could still contribute to the DNA pool from which whole-community amplicons were produced.

The values recorded for the basic physicochemical variables of the cheeses were broadly consistent with previously reported results for Cabrales [16] and similar to those noted for other blue-veined cheeses [34,35,36]. Therefore, Cabrales cheese’s physicochemical framework would seem to be rather constant and well-defined. Amino acids and amino-acid-derived compounds in cheese are associated with aroma and taste [46]. Free amino acids steadily increase during cheese aging; indeed, given the extensive proteolytic activity of *P. roqueforti* in blue-veined cheeses, free amino acids and amino-derived compounds are to be expected in abundance in long-ripened cheeses [36]. Glutamic acid, leucine, isoleucine, proline, and lysine have all been reported in large quantities in cheese, reflecting the amino acid composition of milk caseins [8,37]. The specific concentrations of amino acids in the present cheeses were similar to those previously reported for other blue-veined varieties [35] and other long-ripened cheese types, such as Gouda [37] and Grana Padano [47].

Biogenic amines are toxic compounds formed by the microbial decarboxylation of amino acids, which can accumulate in cheese [48]. These may have adverse health effects, and some (e.g., putrescine, cadaverine) may lead to the development of off-flavours [49]. Moderate-to-high concentrations of several biogenic amines were recorded for the present cheeses, as previously reported for Cabrales [50]. However, in Cabrales cheese, only histamine and tyramine might reach levels of potential health concern. The small portions of Cabrales cheese commonly consumed make intoxication by biogenic amines unlikely (limited exposure). Nonetheless, sensitive consumers, or those under treatment with monoaminoxidase (MAO) inhibitors, should avoid eating aged cheeses. Among the amino acid derivatives, high concentrations of GABA were detected in two cheeses. GABA is produced by the decarboxylation of glutamate and is thought to be of benefit to health and well-being by reducing stress, improving sleep, modulating kidney function, and lowering blood pressure [51]. It can, however, also lead to cheese texture defects, as recently reported for Gouda [37]. In the latter work, GABA production was associated with the excessive development of *L. rennini*, a bacterium determined to be highly abundant in Cabrales. The connection between *L. rennini* and GABA in the cheeses of this study was further confirmed by correlation analysis, which indicated a strong, direct association between this bacterium and the compound.

The Cabrales cheese matrix was depleted of lactose (only about 0.6 g 100 g^−1^ was present), glucose, and galactose, similar to that reported for other long-ripened cheeses [34,37]. Compared to fermented milk [52] and short-ripened cheeses [8], the content of other organic acids, such as formic, orotic, pyruvic, and uric acids, was low in the present Cabrales cheeses. Lactic, acetic, and butyric acids were the only organic compounds detected in sufficient enough quantities to influence the sensory profile. Certainly, lactic acid is responsible for the sour taste of cheeses and has recently been associated with sweet and umami tastes [53]. Acetic and butyric acids, which can be formed during bacterial fermentation but also from the catabolism of fatty acids [46], further contribute to an acidic taste. The significant positive correlation between *L. lactis* (which produces lactic acid) and *P. roqueforti* (which requires lactic acid) may rely on the nutritional dependence of the latter upon the main metabolic end product of the former. The nature of other interactions detected by correlation analysis between the microbial populations deserves further investigation.

VOCs provide the typicity and authenticity of cheeses and can be used as tracers of the ripening process [53]. However, the use of different analytical methods and equipment makes it difficult to compare results across studies [7,37,54,55]. VOCs may have their origin in microbial activity involving lactose and milk proteins and fatty acids [49]. Most compounds, however, appear as a result of proteolysis, lipolysis, and amino acid and fatty acid catabolism [46]. Data on the aroma notes, odour intensity, and VOC detection thresholds in dairy products have been recently reviewed [7,53]. Several carboxylic acids (pungent flavours), alcohols (sweet, honey, flower), esters (fruity, sweet, creamy notes), and ketones (mushrooms, yogurt, and creamy odours) make up the majority of VOCs in Cabrales. The large variation in the content of these compounds among the present cheeses may partially result from compositional differences in the milk (different mixtures of cow’s, sheep’s, and goat’s milk). The complexity of VOCs in both number and concentration and the interaction between components impede an accurate identification of the key aroma compounds in Cabrales. As suggested by statistical analyses, the sensory profiles of the cheese may rely on a balanced concentration of several chemical compounds, among which butyric acid and lactic acids might be pivotal. Furthermore, the relative abundance of *Tetragenococcus* and *Staphylococcus* species and the correlation between these bacteria and majority metabolites in Cabrales suggests species of these two genera may have a significant role in the sensory notes of the cheese.

## 5. Conclusions

In this study, 16 high-quality, long-ripened Cabrales cheeses were microbiologically and biochemically characterised. The NGS microbial analysis revealed a remarkable microbial diversity, although significant differences in both prokaryotic and eukaryotic populations were found between cheese samples in terms of both numbers and biotypes. Species of *Tetragenococcus, Lactococcus*, and *Staphylococcus* genera were the majority of bacteria, followed by those of *Brevibacterium* and *Corynebacterium*. The starter *P. roqueforti* was the most abundant eukaryotic organism in all samples, followed by yeast species of the genera *Debaryomyces, Geotrichum*, and *Kluyveromyces*. Complex biochemical profiles were obtained, particularly for organic acids, free amino acids, and VOCs; of these, free amino acids were the most abundant metabolites and can be used as markers of Cabrales cheese ripening. The knowledge gathered in this work may serve for the development of synthetic starter mixtures, which might contribute towards increasing Cabrales cheese quality. A complex mixture of bacterial (*L. lactis*, *T. koreensis*, *S. equorum*, *B. linens*, and *C. variabile*) and fungal (*P. roqueforti*, *D. hansenii*, *G. candidum*, and *K. lactis*) strains is proposed (and planned to be tested) as a microbiota-based starter. The use of *L. rennini* as an adjunct culture to enhance the concentration of GABA in Cabrales cheese deserves further investigation.

The present work suffers from the relatively low number of samples analysed, and the majority of the examined cheeses were likely manufactured during the same season (winter 2021–2022). Analysing more samples from more producers and of cheeses manufactured throughout the year would be beneficial. Complementing amplicon sequencing with shotgun metagenomic analyses would more precisely identify the microbial diversity in Cabrales cheese. Monitoring the microbial quality of ingredients (milk, rennet, and salt), manufacturing settings and equipment (air, vats, moulds, shelves), and ripening environment (caves) would provide information on the sources of the microorganisms, information that could ultimately be used uncover the interrelationships between microbes and cheese safety and quality.

## Figures and Tables

**Table 1:** Viable counts (log_10_ CFU/g) of presumptive microbial groups in the Cabrales cheese samples of this study.

Microbial Group (Counting Medium)	Cheese Sample															
	C1	C2	C3	C4	C5	C6	C7	C8	C9	C10	C11	C12	C13	C14	C15	C16
Total viable mesophilic counts (PCAM)	5.97 ^a^	5.56	5.00	4.51	2.00	5.42	6.95	3.56	7.39	5.53	6.47	5.46	5.38	7.65	7.14	4.94
Lactococci/streptococci (GM17A)	4.63	4.67	3.55	4.34	2.60	5.26	4.65	2.30	5.49	3.60	5.27	3.04	4.06	7.16	4.89	3.00
*Lactobacillaceae* (MRSA)	4.70	4.58	2.78	3.96	<2 ^b^	4.43	<2	2.60	5.31	<2	5.11	2.48	3.27	6.70	4.69	2.30
Enterococci (S-BA)	3.68	4.71	<2	4.17	<2	4.57	5.30	2.00	3.31	4.23	4.86	2.90	4.11	4.46	3.65	2.00
Micrococci/staphylococci (B-PA)	<2	4.54	3.72	4.37	<2	3.18	4.31	<2	5.68	4.41	4.71	2.85	3.30	6.18	3.45	<2
*Enterobacteriaceae* (VRBGA)	<2	<2	<2	<2	<2	<2	<2	<2	<2	<2	<2	<2	<2	<2	<2	<2
Coliforms (VRBLA)	<2	<2	<2	<2	<2	<2	<2	<2	<2	<2	<2	<2	<2	<2	<2	<2
Yeasts and moulds (YGCA)	6.79	3.85	6.35	4.94	2.60	6.52	6.42	5.11	4.61	6.47	6.08	6.15	4.26	7.31	7.12	4.00

^a^ Data are expressed as mean values of two counting plates containing between 30 and 300 colonies. ^b^ Counts lower than the limit of detection (10^2^ CFU/g of cheese).

**Table 2:** Content of organic acids, sugars, and other compounds (mg 100 g^−1^) in the Cabrales cheese samples of this study.

Cheese Sample	Organic Acid/Compound											
	Acetic ^a^	Butyric	Formic	Lactic	Orotic	Pyruvic	Uric	Lactose	Glucose	Galactose	Ethanol	Ammonia
C1	199	95	-	397	3	16 ^b^	10	714	24	-	-	4590
C2	201	209	-	125	1	8	13	566	22	-	36	4016
C3	385	326	-	247	1	5	4	562	26	17 ^b^	101	1505 ^b^
C4	322	430	14 ^b^	422	1	7	18	707	4	-	105	3989
C5	381	373	21 ^b^	351	1	9	12	536	6	-	-	4569
C6	227	454	-	405	2	11	24	598	11	7 ^b^	-	4036
C7	91	66	-	187	2	17 ^b^	21	446	13	-	-	5104
C8	360	431	-	234	1	11	19	588	9	7 ^b^	22	4009
C9	390	345	-	190	3	1	13	583	4	-	148	4753
C10	352	199	-	140	-	9	14	421	4	-	120	3802
C11	220	206	-	70	3	6	24	517	5	-	-	3448
C12	96	136	-	199	1	7	21	540	13	5 ^b^	-	3662
C13	349	308	-	165	1	4	8	597	2	-	100	3507
C14	178	105	-	14	1	3	21	422	10	-	81	3696
C15	23	142	-	99	1	7	11	473	-	-	82	2997
C16	388	125	-	169	1	7	12	451	-	-	120	4248

^a^ The concentration of acetic acid is estimated as this compound co-elutes with uric acid. ^b^ Outliers determined by the interquartile range (IQR) method. -, not detected.

**Table 3:** Content of biogenic amines and decarboxylated amino acids (mg 100 g^−1^) in the Cabrales cheese samples of this study.

Cheese Sample	Compound									
	Agmatine	Cadaverine	Ethylamine	Histamine	Ornithine	Putrescine	Tyramine	Tryptamine	β-Phenylethylamine	GABA
C1	-	58	-	40	237	8	279	82	7	1337 ^a^
C2	-	73	-	7	161	2	172	65	9	235
C3	-	37	-	14	197	13	245	-	5	9
C4	-	39	-	85	268	12	290	25	5	313
C5	-	118	2 ^a^	-	317	1	513	144	14	16
C6	-	152	2 ^a^	74	211	22	508	139	18	392
C7	11 ^a^	247	-	-	186	10	145	174	30	15
C8	9	169	-	-	224	3	130	64	20	74
C9	3	31	-	60	423	1	457	-	4	5
C10	-	9	-	-	322	-	186	61	1	354
C11	4	251	-	27	161	68 ^a^	412	110	30	213
C12	4	121	-	13	132	7	220	107	15	129
C13	4	59	-	70	298	2	302	29	7	637
C14	-	6	-	-	244	-	225	11	1	5
C15	-	173	-	-	174	-	145	3	21	9
C16	-	341	-	-	158	11	224	116	41	20

GABA, gamma-aminobutyric acid. ^a^ Outliers determined by the interquartile range (IQR) method. -, not detected.

## Data Availability

The raw and assembly metataxonomic data obtained in this study were deposited in the Sequence Read Archive (SRA) of the NCBI database (http://www.ncbi.nlm.nih.gov, accessed on 6 November 2024) under the BioProject PRJNA1182749.

## Supplementary Materials

The following supporting information can be downloaded at: https://www.mdpi.com/article/10.3390/foods14132366/s1, Table S1: Amino acid content (mg 100 g^−1^) in the ripened Cabrales cheeses of this study; Table S2: Relative abundance of the volatile compounds detected in the Cabrales cheeses of this study.
