# Supplementary material for: Microbial and Biochemical Analyses of High-Quality, Long-Ripened, Blue-Veined Cabrales Cheese
Source: Foods. 2025 Jul 3;14(13):2366. doi: 10.3390/foods14132366 (PMC12248881; doi:10.3390/foods14132366)
Supplement: Supplementary file 1 [file foods-14-02366-s001.zip › foods-3707248-supplementary.pdf]

Table S1.- Amino acid content (mg 100 g^{-1}) in the ripened Cabrales cheeses of this study.

Cheese sample	Amino acid[a]																			
	**Ala**	**Arg**	**Asn**	**Asp**	**Cys**	**Gln**	**Glu**	**Gly**	**His**	**Ile**	**Leu**	**Lys**	**Met**	**Phe**	**Pro**	**Ser**	**Thr**	**Trp**	**Tyr**	**Val**
**C1**	805	88[b]	66	332	7	231	777	302	554	921	1388	1608	451	798	20	166	288	135	438	1131
**C2**	917	53	24	827	10	88	2388	254	467	660	1022	1343	366	692	33	307[b]	237	110	332	998
**C3**	276[b]	96[b]	40	309	8	243	834	89	172	397	493	599	353	263[b]	72	129	256	128	248	355[b]
**C4**	617	17	8	504	6	45	1788	181	297	498	683	1307	282	492	64	61	50	80	96	742
**C5**	637	31	17	319	7	149	2200	148	264	623	1049	1270	323	636	21	78	92	51	225	941
**C6**	655	33	40	796	10	246	1941	243	405	690	1085	1267	388	671	43	177	283	31	105	964
**C7**	992	42	10	864	10	175	2344	304	394	834	1301	1051	424	819	22	85	68	57	881	1029
**C8**	817	54	11	860	13	633	2057	253	386	821	1337	1105	469	793	30	370[b]	253	112	739	1024
**C9**	842	34	102[b]	1275	5	522	3234[b]	432[b]	569	837	1398	1804	522	869	-	637[b]	466	210[b]	128	1285[b]
**C10**	730	52	29	759	6	415	1593	225	403	682	1187	1513	425	664	58	121	116	81	341	958
**C11**	730	19	5	276	5	30	1908	172	298	748	1299	860	369	618	73	118	101	35	105	847
**C12**	888	75	17	753	9	500	1779	218	375	678	1163	1186	373	694	45	193	156	54	545	965
**C13**	819	27	23	632	6	16	1592	162	339	590	1043	1317	366	631	71	152	144	106	163	923
**C14**	476	52	34	761	3	347	1949	225	473	565	830	1395	290	517	78	104	126	73	157	759
**C15**	292[b]	34	61	494	5	341	1462	103	312	508	775	1010	288	487	123	179	184	80	176	597
**C16**	934	36	21	707	3	340	2204	99	368	586	968	858	288	697	35	119	91	47	614	947

[a]Key of amino acids: Ala, alanine; Arg, arginine; Asn, asparagine; Asp, aspartic acid; Cys, cysteine; Gln, glutamine; Glu, glutamic acid; Gly, glycine; His, histidine; Ile, isoleucine; Leu, leucine; Lys, lysine; Met, methionine; Phe, phenylalanine; Pro, proline; Ser, serine; Thr, threonine; Trp, tryptophan; Tyr, tyrosine; Val, valine.
[b]Outliers determined by the Interquartile Range (IQR) method.
-,not detected.

Table S2.- Relative abundance of the volatile compounds detected in the Cabrales cheeses of this study.

Class/compound	Cheese sample															
	C1	C2	C3	C4	C5	C6	C7	C8	C9	C10	C11	C12	C13	C14	C15	C16
**Acids**																
Acetic acid	3.48	6.6	26.7	16.5	25.8	21.3	-	23.2	15.6	11.2	24.6	5.8	18.5	13.1	-	22.0
9-Decenoic acid	-	8.7	7.1	7.6	9.1	8.0	-	9.8	8.0	5.4	6.8	7.8	10.9	2.6	2.9	5.7
Butanoic acid	44.78	-	202.8	-	179.7	230.9	47.9	-	136.9	107.6	145.9	113.9	174.0	76.6	118.8	96.3
Butanoic acid 2-methyl-	-	-	-	-	-	-	53.1*	-	-	-	-	-	-	-	-	148.7*
Butanoic acid 3-methyl-	24.5	70.4	-	-	-	-		-	-	-	64.4	50.1	100.4	85.1	-	-
Dodecanoic acid	3.43	10.0	9.0	9.7	13.8	15.3	5.2	12.6	6.7	7.0	6.6	11.7	12.1	4.9	3.2	5.6
Heptanoic acid	-	8.9	12.8	14.5	13.9	6.0	-	5.6	9.0	5.5	3.0	7.7	10.8	3.9	1.7	4.6
Hexanoic acid	110.7	239.5	276.2	302.1	327.4	306.4	111.7	316.6	253.0	195.4	220.4	205.2	295.2	126.4	164.5	171.9
Hexanoic acid 2-methyl-	-	-	87.6	43.1	127.7	45.8	-	106.0	67.8	83.4	-	-	-	-	75.5	-
Mercaptoacetic acid (2TMS derivative)	-	-	-	-	-	-	-	-	-	1.4	-	-	-	-	-	-
Capric acid	22.8	78.6	75.6	77.2	91.4	90.3	26.8	77.6	64.0	62.5	59.8	77.7	88.0	36.9	38.9	56.7
Nonanoic acid	-	2.6	4.3	4.5	3.3	1.7	-	-	2.3	2.0	1.2	2.8	2.8	1.8	0.8	1.8
Caprylic acid	40.2	136.6	134.7	141.0	164.6	149.3	37.4	153.0	136.2	102.5	110.7	121.5	151.5	58.2	72.4	95.4
Pentanoic acid	-	5.8	15.5*	16.3	11.3	7.9	-	5.0	5.5	4.1	2.8	4.8	7.6	2.7	2.4	4.6
Phosphonic acid (p-hydroxyphenyl)-															0.9*	
Propanoic acid	-	-	-	1.8	2.0	-	-	-	-	-	4.2	-	1.9	1.4	-	3.4
Propanoic acid 2-methyl-	3.7	6.9	11.0	6.1	18.9	5.9	4.7	12.8	9.3	11.7	7.2	5.4	11.3	9.4	9.6	23.0*
Tetradecanoic acid	-	-	-	-	-	2.1*	-	-	-	-	-	-	-	-	-	-
**Aldehydes**																

4-Cyclohexylidenebutyraldehyde	1.3															
**Alcohols**																
(R)-(-)-2-Pentanol	17.6*	-	6.2*	-	-	-	-	16.0*	-	9.2*	-	-	-	-	-	-
1-Propanol 3-(methylthio)-	-	1.8*	1.7*	-	-	-	-	-	-	-	-	-	-	-	-	-
2-Heptanol	113.0*	34.6	30.1	7.4	25.7	79.2	41.5	40.0	36.0	35.7	22.8	23.3	28.8	41.5	18.6	25.3
2-Nonanol	145.1*	30.9	42.3	7.8	40.6	82.9	82.0	61.9	8.1	23.4	9.9	32.4	33.7	30.0	15.3	18.9
2-Undecanol	14.0*	-	-	-	-	2.2*	6.0*	-	-	-	-	-	-	-	-	-
Cyclohexanecarboxylic acid	-	-	-	-	-	-	2.5*	-	-	-	-	-	-	-	-	-
p-Cresol	-	-	-	-	-	-	-	-	-	-	-	-	-	-	1.2*	-
Phenol	-	-	-	-	2.1*	-	-	-	-	-	1.4*	-	-	-	-	-
Phenol 3-methyl-	-	-	-	-	-	3.1*	-	-	-	-	-	-	-	-	-	-
Phenylethyl Alcohol	21.6*	9.0*	2.3	-	2.9	4.3	11.3	-	-	4.0	3.1	4.7	3.7	4.6	2.5	2.7
**Esters**																
2(3H)-Furanone 5-butyldihydro-	3.1	3.4	-	-	1.8	1.6	3.9	3.2	-	2.2	1.5	2.5	-	3.1	1.2	2.2
(-)-1-Methylbutyl decanoate	-	3.3*	-	-	-	-	-	-	-	-	-	-	-	-	-	-
1.3-Dioxolane-4- methanol 2-pentadecyl-acetate cis-	-	-	4.2*	-	-	-	-	-	-	-	-	-	-	-	-	-
1.3-Dioxolane-4-methanol 2-pentadecyl-acetate trans	-	-	-	-	-	-	-	6.2*	-	-	-	-	-	-	-	-
2(3H)-Furanone 5-ethyldihydro-	17.1	17.8	7.7	3.3	9.0	12.6	22.9	16.5	4.1	12.5	10.9	16.2	5.0	13.5	8.9	14.0
2-Octen-1-ol. 3.7-dimethyl-isobutyrate (Z)-	1.1*	-	-	-	-	-	-	-	-	-	-	-	-	-	-	-
2-Pentanol decanoate	4.7	-	2.0	-	-	5.7*	-	4.3	-	2.5	-	-	-	-	-	-
4-Decenoic acid methyl ester	-	-	-	1.7*	3.4*	2.3*	-	-	-	-	-	-	-	-	-	-
Butanoic acid 1-methylbutyl ester	28.0	22.8	14.3	-	10.6	17.1	-	26.2	8.1	13.3	-	-	-	-	-	-

Butanoic acid 1-methylhexyl ester	13.4*	5.4	-	-	7.0	-	3.1	11.5	-	1.6	-	3.7	4.7	-	1.5	1.8
Butanoic acid 3-methylbutyl ester	16.5	15.2	14.4	2.3	9.3	13.4	6.2	21.0	6.2	14.8	7.6	10.6	6.4	6.1	6.6	7.3
Butanoic acid butyl ester	6.9*	-	-	-	-	-	-	-	-	-	-	-	3.2*	-	-	-
Butanoic acid ethyl ester	-	-	8.7*	19.8*	-	-	-	13.4*	-	-	-	-	-	-	-	-
Butyl 9-decenoate	-	-	-	-	-	1.3*	-	-	-	-	-	-	-	-	-	-
Butyl caprylate	-	-	-	-	-	2.2*	-	-	-	-	-	-	-	-	-	-
Decanoic acid ethyl ester	11.3	16.8	22.5	58.7*	81.7*	68.5*	7.9	24.0	10.3	14.6	12.6	10.7	13.5	3.2	3.6	8.8
Decanoic acid methyl ester	-	-	-	17.1*	-	-	-	-	12.9*	-	-	-	-	-	-	-
Dehydromevalonic lactone	16.4	10.2	8.8	4.1	5.9	9.9	29.8*	17.1	2.4	3.2	3.0	4.0	3.5	6.0	3.1	7.3
Dodecanoic acid methyl ester	-	-	-	-	-	-	2.3*	-	-	-	-	5.0*	-	-	1.4*	-
Ethyl 9-decenoate	-	1.7	2.0	5.3	8.5*	6.5*	-	2.7	-	-	-	-	-	-	-	-
Ethyl 9-tetradecenoate	-	-	-	-	1.2*	-	-	-	-	-	-	-	-	-	-	-
Formic acid, 3-(2-methoxyethyl)-heptyl ester)	-	-	-	-	-	-	-	-	-	7.1*	-	-	-	-	-	-
Heptanoic acid ethyl ester	-	-	-	9.0*	12.4*	-	-	-	-	-	-	-	-	-	-	-
Hexanoic acid 1-methyl hexyl ester	17.0	13.1	12.7	-	14.9	24.1	-	-	3.5	-	-	-	10.3	-	-	-
Hexanoic acid 2-methyl butyl ester	-	-	19.4*	5.0*	-	46.7*	-	-	-	-	-	-	-	-	-	-
Hexanoic acid 2-methyl propyl ester	-	2.2*	-	-	-	2.8*	-	-	-	-	-	-	-	-	-	-
Hexanoic acid 3-pentyl ester	19.1	31.2	-	-	22.2	-	3.6	57.6*	22.7	18.5	5.9	11.3	11.2	-	2.5	4.1
Hexanoic acid butyl ester	1.4	2.7	9.0	5.3	9.7	10.8	-	9.7	-	1.9	-	4.0	8.1	-	1.9	-
Hexanoic acid ethyl ester	15.9	36.9	75.0	162.7*	171.6*	169.7*	6.6	69.7	53.3	33.0	34.8	30.0	36.4	4.6	18.4	19.4
Hexanoic acid methyl ester	-	-	-	33.4*	-	-	-	-	-	-	-	-	-	-	-	-
Hexanoic acid nonyl ester	3.3*	-	-	-	-	-	-	-	-	-	-	-	-	-	-	-
Hexanoic acid propil ester	-	2.0*	-	5.9*	4.2*	4.1*	-	-	-	-	-	-	-	-	-	-

Hexanoic acid undec-10-enyl ester	-	-	-	-	-	-	-	-	-	-	-	11.3*	-	-	-	-
Isopentyl 3-methylpentanoate	-	-	-	-	12.6	17.1	8.3	33.0*	5.6	11.2	6.8	16.2	6.7	-	-	5.2
Isopentyl hexanoate	27.8*	-	-	-	-	-	-	-	-	-	-	-	-	-	-	-
n-Capric acid isopropyl ester	-	-	-	-	-	21.5*	-	-	-	-	-	-	-	-	-	-
n-Octanoic acid isopropyl ester	5.5*	4.2*	-	-	-	-	-	-	8.0*	2.9*	-	-	-	-	-	-
Nonanoic acid ethyl ester	-	-	-	3.4*	-	-	-	-	-	-	-	-	-	-	-	-
Octanoic acid 3-methyl butyl ester	6.4*	2.9	2.0	-	1.5	2.8	1.5	3.6	-	-	-	2.4	-	-	-	-
Ethyl carpylate	11.4	31.7	33.9	95.6*	132.2*	113.0*	5.3	40.1	14.1	22.3	19.9	21.0	19.8	2.1	7.8	8.5
Pentadecanoic acid 3-methyl butyl ester	5.1	2.9	2.6	-	-	2.2	-	3.9	-	-	-	-	-	-	-	-
Propanoic acid 2-amino-3-hydroxy-ethyl ester	-	-	-	-	-	-	-	-	-	-	3.1*	-	-	-	-	-
Tetradecanoic acid ethyl ester	-	-	-	1.4*	1.6*	1.5*	-	-	-	-	-	-	-	-	-	-
**Ketones**																
(Z)-Undec-6-en-2-one[a]	7.0	-	-	-	-	-	7.6	-	-	-	-	-	-	2.2	-	-
(Z)-Undec-6-en-2-one[a]	17.7	3.2	2.3	-	-	-	13.2	4.4	-	2.6	2.8	4.3	-	5.4	-	-
(Z)-Undec-6-en-2-one[a]	31.3*	7.5	2.6	-	-	2.3	24.3*	6.4	-	-	-	14.0	-	9.1	3.8	-
1.4-Cyclooctanedione	-	-	-	-	-	-	-	1.6*	-	-	-	-	-	-	-	-
2-Decanone	24.6*	7.1	5.1	-	2.8	2.2	17.6*	4.9	-	4.8	2.5	12.6	-	15.3	3.1	3.4
2-Heptanone	312.7	204.8	119.8	27.8	72.1	96.2	280.7	210.1	59.3	292.4	221.1	292.6	105.2	332.1	196.9	206.7
2-Heptanone 5-methyl-	-	-	-	3.1*	-	-	-	-	-	-	-	-	-	-	-	-
2-Nonanone	802.8*	349.6	184.2	65.8	145.4	171.8	723.1*	351.3	52.2	313.7	288.7	497.1	122.2	488.8	303.8	243.7
2-Octanone	27.8	17.0	9.2	-	4.1	3.8	25.6	7.3	-	21.7	10.3	29.2	5.5	51.9*	8.6	14.5
2-Pentanone	30.3*	-	-	-	-	-	40.7*	-	-	-	-	-	-	44.6*	3.1*	-
2-Propanone 1-methoxy-	-	-	-	-	-	-	-	-	4.5*	-	-	-	-	-	-	-

2-Tridecanone	15.6*	2.1	3.1	-	-	3.0	11.7*	4.9	-	-	-	2.1	-	1.3	1.4	-
2-Undecanone	282.9*	68.2	56.0	-	39.9	64.7	229.1*	114.5	-	42.9	41.2	93.1	25.3	75.3	49.0	31.5
5-Nonen-2-one[a]	2.4*	-	-	-	-	-	-	-	-	-	-	-	-	-	-	-
5-Nonen-2-one[a]	-	-	-	-	-	-	1.8*	-	-	-	-	-	-	-	-	-
7-Decen-2-one	1.3*	-	-	-	-	-	-	-	-	-	-	-	-	1.0*	-	-
7-Octen-2-one 6-methyl-	3.2*	-	-	-	-	-	2.5*	-	-	-	-	-	-	2.3*	-	-
8-Nonen-2-one[a]	186.7	106.6	27.2	7.9	22.8	21.7	197.3	87.8	7.8	86.7	90.1	151.3	25.9	133.4	76.3	75.6
8-Nonen-2-one[a]	4.9	-	-	-	-	-	1.9	-	-	-	-	-	-	-	-	-
8-Nonen-2-one[a]	5.6	-	-	-	-	-	3.7	-	-	-	-	-	-	-	-	-
Benzyl methyl ketone	-	-	-	-	-	-	-	-	1.4*	-	-	-	-	-	-	-
γ-Chlorobutyrophenone	-	-	-	-	-	-	-	-	-	7.0*	10.0*	-	-	-	-	-
2-Cyclohexen-1-one 3-methyl-	-	-	-	-	-	-	-	-	-	-	-	-	-	2.6*	-	-
**Aromatic compounds**																
Pyrazine tetramethyl-	1.6*	-	-	-	-	-	-	-	-	-	-	-	-	1.1*	-	-
3-Oxo-4-phenylbutyronitrile	-	-	-	-	3.4*	-	-	-	-	5.5*	-	-	-	-	-	-
Benzene (2-methyl-3-butenyl)-	-	-	-	-	-	-	-	4.5*	-	-	-	-	-	-	-	-
Benzene 1-methoxy-4-methyl-	-	-	-	-	-	-	2.4*	-	-	-	-	-	-	-	2.4*	2.3*
Indole	-	1.3	1.5	6.2	16.2*	-	1.7	5.2	-	-	1.6	-	4.5	-	2.3	23.9*
Pyrazine 2.5-dimethyl-	-	-	-	-	-	-	-	-	-	-	3.9*	-	-	-	-	-
Pyrazine trimethyl-	-	-	-	-	-	-	-	-	-	-	-	-	-	6.0*	-	-
**Other**																
9-Decen-1-ol methyl ether	20.0*	-	-	-	-	-	11.6*	-	-	-	-	-	-	5.5*	-	-
4.7-Methanoazulene 1.2.3.4.5.6.7.8-octahydro-1.4.9.9-tetra	-	-	-	-	-	-	9.2*	-	-	-	-	-	-	-	-	-
Albocycline methyl ether	-	-	20.5*	-	-	-	-	-	-	-	-	-	-	-	-	-

Ammonium acetate	-	-	-	-	-	-	11.8*	-	-	-	-	-	-	-	7.9*	-
Heptane 4-ethyl-	2.2*	-	-	-	-	-	-	-	-	-	-	-	-	-	-	-
Melezitose	1.9*	-	-	-	-	-	-	-	-	-	-	-	-	-	-	-
m-Aminophenylacetylene	-	-	-	-	-	-	-	-	-	-	-	0.8*	-	0.7*	-	-

[a]Distinct chromatographic peaks identified as the same compound.

*Outliers detected by IQR analysis.
